# Identification of Novel Therapeutic Agent Candidates Through High Throughput Screening With Chemical Library Based on Molecular Subclassification in Canine Histiocytic Sarcoma Cell Lines

**DOI:** 10.1111/vco.70077

**Published:** 2026-05-13

**Authors:** Hiroki Sakuma, Hirotaka Tomiyasu, Mari Takamiya, Yuko Goto‐Koshino, Atsushi Tsukamoto, Makoto Bonkobara, Masaru Okuda

**Affiliations:** ^1^ Laboratory of Veterinary Internal Medicine, Veterinary Clinical Science Unit, Department of Veterinary Medical Sciences, Graduate School of Agricultural and Life Sciences The University of Tokyo Bunkyo Tokyo Japan; ^2^ Drug Discovery Initiative, Graduate School of Pharmaceutical Sciences The University of Tokyo Bunkyo Tokyo Japan; ^3^ Veterinary Medical Center, Graduate School of Agricultural and Life Sciences The University of Tokyo Bunkyo Tokyo Japan; ^4^ Laboratory of Laboratory Animal Science Azabu University Sagamihara Kanagawa Japan; ^5^ Department of Veterinary Clinical Pathology Nippon Veterinary and Life Science University Musashino Tokyo Japan

**Keywords:** Akt pathway, dog, duvelisib, molecular‐targeted drug, PI3Kγ

## Abstract

Effective chemotherapy for canine histiocytic sarcoma (CHS) has yet to be established. In our previous study, CHS cell lines were subclassified into two groups based on their gene expression profiles: Group A and Group B. This study aimed to identify novel therapeutic agents that are effective against each CHS subgroup, and we performed high throughput screening, which examined the effects of 1824 chemical compounds on the 10 CHS cell lines. Among the compounds examined, 73 compounds were identified as candidates to show antiproliferative effects in all CHS cell lines examined, and the median IC_50_ values of 5‐fluorouracil and sunitinib were 7.49 μM and 608 nM, respectively, across all CHS cell lines. While there was no compound that was specifically effective for Group B CHS cell lines, duvelisib was identified as a selective antitumor agent for CHS cell lines in Group A (median IC_50_ = 287 nM), with minimal effects on the other group (median IC_50_ > 5 μM) and normal peripheral blood mononuclear cells (IC_50_ = 7.46 μM). The amount of phosphorylated Akt protein decreased following duvelisib treatment in all eight CHS cell lines in Group A, but not in cell lines in Group B. Furthermore, duvelisib induced cell cycle arrest and increased cell death in a subset of Group A cell lines. These findings demonstrated that duvelisib exerted antiproliferative effects through inhibition of the Akt pathway in a subset of CHS cell lines while causing minimal cytotoxicity to normal cells.

## 1 Introduction

Canine histiocytic sarcoma (CHS) is a rare and highly aggressive tumour originating from interstitial dendritic cells and macrophages [1]. It can arise in various organs, including bone marrow, lungs, skin, and spleen [1]. Current treatment options include surgery, radiation therapy, and chemotherapy [2]. Among these, chemotherapy is generally considered the first‐line treatment; however, many chemotherapeutic agents including lomustine have demonstrated antitumor efficacy in limited cases, resulting in a median survival time of approximately 100 days [3, 4, 5, 6, 7, 8, 9]. Therefore, there is an urgent need to develop more effective therapeutic strategies for CHS.

In human histiocytic neoplasms, the presence of the BRAF V600E mutation and subsequent activation of the ERK signalling pathway have been well documented, with combination therapy using trametinib, a MEK inhibitor, and dabrafenib, a BRAF V600E specific inhibitor, demonstrating superior efficacy relative to other treatment modalities [10, 11, 12]. In CHS, ERK and Akt pathways are commonly activated [13], and previous studies have identified dasatinib, a Src kinase inhibitor, and trametinib as potential therapeutic agents [14, 15, 16, 17]. However, the antiproliferative effects of these inhibitors vary across individual CHS cell lines [18]. To investigate the mechanisms underlying these variabilities in drug sensitivities, we recently performed a comprehensive gene expression analysis of CHS cell lines. Our results showed that CHS cell lines could be subclassified into two groups, Group A and Group B, based on their gene expression profiles, particularly with respect to NF‐κB pathway activation status [19]. However, sufficient antiproliferative effects were not observed, even in cell lines with activated NF‐κB signalling. These findings suggest that the NF‐κB pathway itself may not represent an effective therapeutic target in CHS cells, highlighting the need to identify novel candidate drugs tailored to each molecular subgroup.

In recent years, therapeutic drug discovery in human medicine has significantly advanced through high throughput screening (HTS) using large compound libraries [20]. In CHS, compounds such as dasatinib and trametinib have also been identified via examinations using compound libraries [14, 17], but their scales were relatively small. In addition, considering the highly variable responses to antiproliferative drugs among CHS cell lines, screening for effective antitumor agents should be conducted based on the molecular subgroups of CHS cells rather than focusing on specific drugs. Therefore, the present study performed HTS using a broad range of compounds on CHS cell lines to identify highly effective antiproliferative agents for each molecular subgroup.

## 2 Materials and Methods

### 2.1 Cell Lines and Cell Culture

Ten CHS cell lines were used in this study. CHS2, CHS4, CHS5, CHS6, CHS7, CHS8 (ROMA), and MHT2, were previously established at Nippon Veterinary and Life Sciences University [21], while DHS1 and DHS2 were established at the University of Tokyo [13]. The DH82 cell line was originally derived from a dog with hemophagocytic CHS [22]. These 10 cell lines were also used in our previous studies [13, 18, 19]. The two subgroups based on gene expression profiles were designated as Group A (CHS2, CHS4, CHS5, CHS6, CHS7, CHS8, DHS2, and MHT2) and Group B (DH82 and DHS1) (Table 1) [19].

**TABLE 1:** Canine histiocytic sarcoma cell lines used in this study.

Cell lines	Breed	Age (years)	Sex	Diagnosis	Biopsy site	Molecular subgroup
CHS2	Shetland sheepdog	8	Male	Disseminated HS	Skin	A
CHS4	Flat coated retriever	6	Female	Solitary HS	Synovial	A
CHS5	Golden retriever	8	Male	Solitary HS	Synovial	A
CHS6	Bernese mountain dog	7	Male	Solitary HS	Lymph node	A
CHS7	Flat coated retriever	8	Male	Solitary HS	Synovial	A
CHS8 (ROMA)	N/A	N/A	N/A	N/A	Skin	A
DH82	Golden retriever	10	Male	Disseminated HS	Spleen	B
DHS1	Shiba‐dog	12	Female	Disseminated HS	Mandible	B
DHS2	Miniature schnauzer	6	Female	Disseminated HS	Pleural effusion	A
MHT2	N/A	N/A	N/A	N/A	N/A	A

*Note:* N/A; Not available.

All cell lines were cultured at 37°C in Dulbecco's Modified Eagle's Medium (DMEM, FUJIFILM Wako Pure Chemical Corporation, Osaka, Japan) supplemented with 10% fetal bovine serum (Biowest, Nuaillé, France) and 1% penicillin–streptomycin (FUJIFILM Wako Pure Chemical Corporation) in a humidified atmosphere containing 5% CO_2_.

### 2.2 Cell Line Authentication Statement

CHS2 to CHS7 were authenticated as CHS cell lines as previously described [21]. The remaining cell lines were confirmed as being derived from CHS through immunohistochemical staining of the original lesions. All CHS cell lines used in this study were confirmed to be free of mycoplasma contamination, as verified by a PCR‐based Mycoplasma Detection Kit (TaKaRa Bio Inc. Shiga, Japan).

### 2.3 Isolation of Peripheral Blood Mononuclear Cells (PBMCs)

Peripheral blood was collected from five healthy dogs maintained in our laboratory and PBMCs were isolated using a standard protocol. Briefly, the cells in the buffy coat were isolated after centrifugation at 800 × g for 10 min, diluted with phosphate‐buffered saline (PBS), layered onto Lymphoprep (STEMCELL Technologies Inc., Vancouver, Canada), and centrifuged at 830 × g for 25 min to isolate PBMCs. All animal procedures were conducted in accordance with the institutional guidelines for animal care and use and approved by the University of Tokyo (Approval No. P21‐140).

### 2.4 HTS Using a Compound Library

A validated library consisting of 1824 compounds, which are already in use or perform clinical trials in human medicine, was provided by the Drug Discovery Initiative at the University of Tokyo. CHS cells and PBMCs were seeded into 384 or 96‐well plates at the concentrations of 1–10 × 10^4^ cells/mL according to the doubling time of each cell line and cultured for 72 h at 37°C in appropriate media containing each compound dissolved in dimethyl sulfoxide (DMSO). CHS cell lines were cultured in DMEM, while PBMCs were cultured in Roswell Park Memorial Institute 1640 medium (RPMI‐1640, Sigma Aldrich, St. Louis, MO). The final concentration of DMSO in the growth medium was maintained at or below 0.25% (v/v), and the same concentration of DMSO was added to control wells. Following the incubation period, 3 μL (for 384‐well plate) or 10 μL (for 96‐well plate) of Cell Counting Kit‐8 (Dojindo Laboratories, Kumamoto, Japan) was added to each well, and the plates were incubated for an additional 3 h at 37°C. Absorbance was measured at 450 nm using a PHERAstar Plus (BMG Labtech, Ortenberg, Germany) for 384‐well plate or a Model 680 Microplate Reader (Bio‐Rad Laboratories, Hercules, CA) for 96‐well plate. Viable cell numbers were calculated based on absorbance readings.

At first, the quality of the screening in each cell line was evaluated based on *Z*' factor, which was calculated by the following formula: *Z*' = 1−(3 × SD_H_ + 3 × SD_L_)/(Av_H_−Av_L_) [23]. SD_H_ means the standard deviation of absorbance values in control wells, SD_L_ means the standard deviation of absorbance values in blank wells, Av_H_ means the average of absorbance values in control wells, and Av_L_ means the average of absorbance values in blank wells. *Z*' factor for each cell line was calculated using the data obtained in six plates for each cell line during primary screening described below, and the quality of screening was validated by confirming that the average of *Z*' factor in each cell line was 0.5 or higher.

The flowchart illustrating the selection process of candidate compounds is shown in Figure S1. As primary screening, we administered 5 μM of all 1824 compounds to each CHS cell line as a single experiment, and the compounds that reduced viable cell count to ≤ 70% were selected as HIT compounds in each cell line. Among these HIT compounds, we included those reducing viable cell count to ≤ 70% in at least two cell lines and excluded the compounds that were previously shown to have antitumor effects in CHS cell lines. Viable cell count was additionally examined twice for the included HIT compounds, and those reducing viable cell count to ≤ 70% at least once in each cell line in these additional experiments were selected as candidate compounds. We further selected the compounds that should be focused using the following criteria: those that reduced viable cell count to ≤ 70% in at least five Group A CHS cell lines and in none of the Group B CHS cell lines (Group A‐specific compound candidates), and those that reduced viable cell count to ≤ 70% in five or fewer Group A CHS cell lines and in all two Group B cell lines (Group B‐specific compound candidates). Then, we administered the compounds that met each of the criteria to all CHS cell lines at various concentrations to calculate the half‐maximal inhibitory concentration (IC_50_) value in each cell line.

### 2.5 Western Blotting

Cellular proteins of CHS cell lines with or without treatment with 500 nM of duvelisib (Selleck Chemicals, Houston, TX) for 1 h were extracted using radio‐immunoprecipitation assay buffer containing 50 mM Tris–HCl, 150 mM NaCl, 0.2 mM EDTA, 1% NP‐40, 1% sodium‐deoxycholate, and 0.1% sodium dodecyl sulfate (SDS) supplemented with 1% protease inhibitor (P8340; Merck Millipore, Burlington, MA) and 1% phosphatase inhibitor cocktail (PhosSTOP; Sigma‐Aldrich). Protein concentrations were measured using the DC Protein Assay Kit (Bio‐Rad Laboratories).

SDS‐polyacrylamide gel electrophoresis was performed using 10 μg of total proteins per sample, followed by transfer to a polyvinylidene difluoride membrane (Merck Millipore). Membranes were blocked with Tris‐buffered saline supplemented with 0.1% Tween‐20 and 4% (w/v) bovine serum albumin (Sigma‐Aldrich), then incubated with primary antibodies (Table S1) overnight at 4°C. After washing, the membrane was incubated with secondary antibodies (Table S1) for 2 h at room temperature. Protein bands were detected using the Immobilon Forte Western HRP substrate (Merck Millipore) and visualised with a ChemiDoc XRS Plus imaging system (Bio‐Rad Laboratories).

### 2.6 Cell Cycle Assay and Measurement of Proportions of Dead Cells

Cells, either untreated or treated with 500 nM of duvelisib (Selleck Chemicals) for 24 h, were collected and fixed in 70% ethanol at −20°C overnight. After fixation, the cells were incubated with RNase (100 μg/mL; Sigma‐Aldrich) for 30 min followed by staining with propidium iodide (PI; 2 μg/mL; Sigma‐Aldrich) for 15 min. Intracellular PI fluorescence was measured using a FACSVerse flow cytometer (BD Biosciences, Franklin Lakes, NJ). The proportions of cells in each phase of the cell cycle were analysed using BD FACSuite software (BD Biosciences).

For the measurements of the proportions of dead cells, cells untreated or treated with 500 nM of duvelisib (Selleck Chemicals) for 48 h were collected and incubated with PI (0.25 μg/10^6^ cells; Sigma‐Aldrich) for 15 min. PI‐positive cells were identified as dead cells using a FACSVerse flow cytometer (BD Biosciences), and their proportions were measured using BD FACSuite software (BD Biosciences).

### 2.7 Reverse Transcription‐Quantitative Polymerase Chain Reaction (RT‐qPCR)

Total RNAs were extracted from the cell lines and PBMCs of five healthy dogs using AllPrep RNA/DNA Mini Kit (Qiagen, Hilden, Germany) according to the manufacturer's instructions. RNA samples were immediately frozen and stored at −80°C until use.

For gene expression analysis using RT‐qPCR, the extracted total RNAs were reverse transcribed using the ReverTra Ace RT Master Mix (Toyobo Co. Ltd., Osaka, Japan) according to the manufacturer's instructions. qPCR was performed using PowerUp SYBR Green Master mix (Applied Biosystems, Foster City, CA) on a StepOnePlus Real‐Time PCR system (Applied Biosystems) using primer pairs listed in Table S2. The gene expression levels of *PI3KCG* and *PIK3CD* genes were relatively quantified using the standard curve method, with *RPL32* used as an internal control, as previously described [13]. All reactions were performed in duplicate.

### 2.8 Cell Viability Measurements After Treatment With PI3Kγ Specific Inhibitor

CHS cells were seeded into 96‐well plates at the concentrations of 1–10 × 10^4^ cells/mL according to the doubling time of each cell line and cultured for 72 h at 37°C in appropriate media containing various concentrations (0.1 nM to 10 μM) of a PI3Kγ‐specific inhibitor (AS‐041164) [24] (Selleck Chemicals) dissolved in DMSO. The final concentration of DMSO in the growth medium was maintained at or below 0.25% (v/v), and the same concentration of DMSO was added to control wells. Cell viabilities were measured using the same methods as in the HTS experiments.

### 2.9 Statistical Analysis

Statistical analyses were performed using R software (ver. 4.3.3; https://www.R‐project.org/). Differences in the proportions of G0/G1 phase cells and dead cells between drug‐treated and untreated conditions in each cell line were assessed using Student's *t*‐test. Differences in the relative amounts of mRNAs of genes were assessed among Group A, Group B, and PBMCs using the Steel–Dwass test. *p* < 0.05 was considered statistically significant.

## 3 Results

### 3.1 HTS With an 1824 Compounds Library in CHS Cell Lines

**FIGURE 1:**
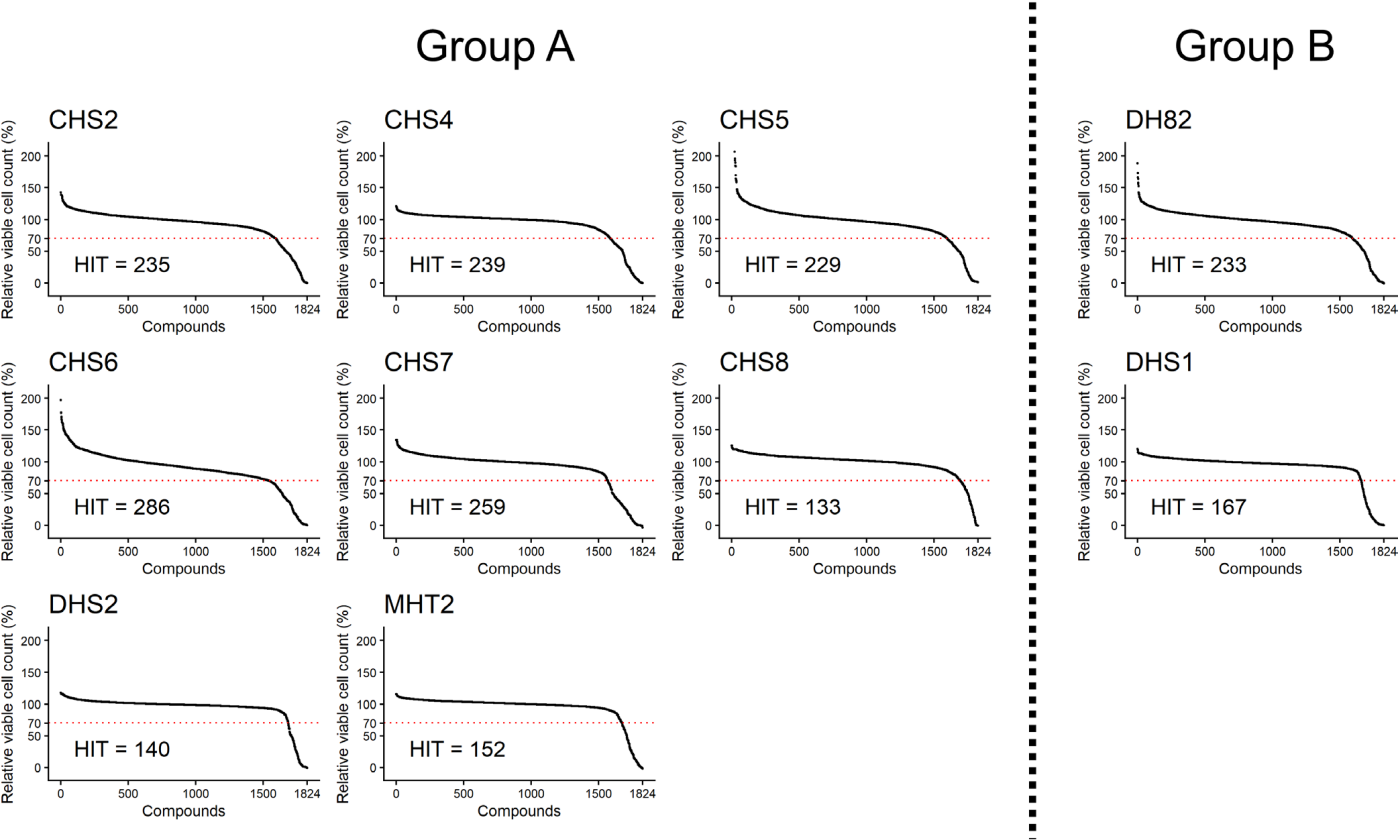
Cell viability obtained by 5 μM of each compound in each canine histiocytic sarcoma cell line (*n* = 1). Each dot represents the relative viable cell count after treatment to the indicated cell line with each compound at a concentration of 5 μM. Compounds are arranged in descending order of relative cell viability after treatment with each compound. “HIT” indicates the number of compounds that reduced viable cell count to ≤ 70% in each cell line.

At first, proliferation inhibition rates of all 1824 compounds at 5 μM were investigated in each cell line as a single experiment (Figure 1). Among the HIT compounds in each cell line, we selected those that reduced viable cell count to ≤ 70% in at least two CHS cell lines, and we excluded dasatinib, trametinib, ponatinib, palbociclib, melphalan, methotrexate, vincristine, vinblastine, paclitaxel, doxorubicin, etoposide, and vinorelbine, because they had been previously reported to have antiproliferative effects on CHS cells and cases [4, 13, 14, 16, 17, 25, 26, 27]. As a result, 299 HIT compounds were included, and viable cell count following treatment was examined in additional duplicate experiments; ultimately, 287 compounds were selected as candidate compounds (Table S3). We found that 73 of the 287 candidate compounds—including 5‐fluorouracil and sunitinib, as well as compounds that exert nonspecific physical or chemical effects on cell membranes—reduced viable cell counts to ≤ 70% in all CHS cell lines (Table 2). The median IC_50_ values were 7.49 μM (range: 42.2 nM—45 μM) for 5‐fluorouracil and 608 nM (range: 70.4 nM—2.3 μM) for sunitinib, respectively. No significant differences in IC_50_ values were observed between Groups A and B for either drug (Figures S2A and S2B). Considering the reported *C*_max_ values after administration at clinical dosages of 5‐fluorouracil in dogs (approximately 700 μM) and sunitinib in humans (approximately 100 nM) [28, 29], IC_50_ values below these concentrations were observed in all CHS cell lines for 5‐fluorouracil, but only in two cell lines (CHS6 and CHS7) for sunitinib.

**TABLE 2:** Compounds which reduced the viable cell counts to ≤ 70% at 5 μM in all canine histiocytic sarcoma cell lines examined with reproducibility in this study.

5‐Fluorouracil
Abemaciclib methanesulfonate
Aclarubicin
Afatinib dimaleate
Almonertinib
Amsacrine hydrochloride
Auranofin
Avapritinib
Belotecan hydrochloride
Belumosudil
Cabazitaxel
Carfilzomib
Ceritinib
Chlorhexidine
Clofarabine
Cobimetinib
Colchicine
Crizotinib
Cycloheximide
Dacomitinib
Dactinomycin
Daunorubicin hydrochloride
Digoxin
Disulfiram
Docetaxel
Ebastine
Elacestrant dihydrochloride
Emetine 2HCl
Entrectinib
Epothilone B
Epothilone D
Exatecan mesylate
Fedratinib
Fenbendazole
Floxuridine
Flubendazole
Homoharringtonine
Idarubicin
Itraconazole
Ixabepilone
Ixazomib citrate
Lanatoside C
Lestaurtinib
Lomitapide
Mebendazole
Mitomycin C
Mitoxantrone dihydrochloride
Mozavaptan
Niclosamide
Olanexidine hydrochloride semihydrate
Olverembatinib dimesylate
Ouabain
Panobinostat
Podophyllotoxin
Proscillaridin A
Pyrvinium pamoate
Quinacrine dihydrochloride dihydrate
Rapamycin
Remogliflozin etabonate
Resibufogenin
Retaspimycin hydrochloride
Rubitecan
Selinexor
Sunitinib
Teniposide
Terfenadine
Thonzonium bromide
Tilorone dihydrochloride
Tirbanibulin
Topotecan
Trabectedin
Tucidinostat
Vorinostat

**FIGURE 2:**
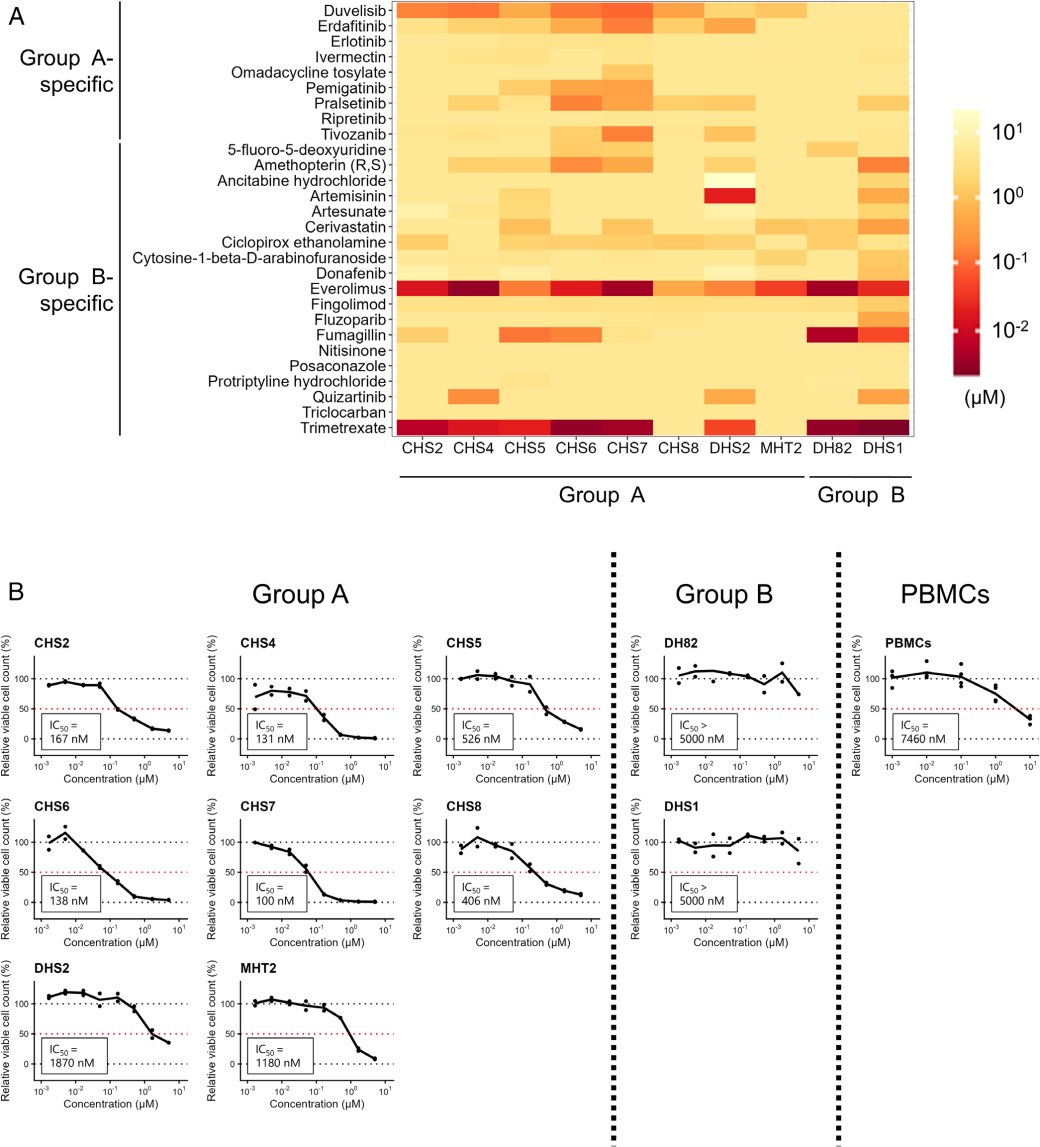
(A) Heatmap showing the half‐maximal inhibitory concentration (IC_50_) values of 28 compounds identified as Group A‐ and Group B‐specific compound candidates in each cell line. (B) Dose–response curves to show antiproliferative effects of duvelisib in each cell line and peripheral blood mononuclear cells (PBMCs) isolated from healthy dogs. IC_50_: Half‐maximal inhibitory concentration.

Since the aim of this study included the identifications of the effective antiproliferative agents for each molecular subgroup, we selected Group A‐ and Group B‐specific compound candidates according to the criteria described above. As a result, 9 compounds were selected as Group A‐specific compound candidates, and 19 compounds were selected as Group B‐specific compound candidates (Table 3). The antiproliferative effects of these 28 compounds were then assessed by determining IC_50_ in each CHS cell line (Figure 2A). Among the selected 9 compounds as Group A‐specific compounds, duvelisib exhibited the antiproliferative activity in Group A CHS cell lines (median IC_50_ = 287 nM [range: 100 nM—1.87 μM]), but not in Group B cell lines (median IC_50_ > 5 μM) (Figure 2B) with the greatest difference in IC_50_ between Groups A and B. The mean IC_50_ of duvelisib for the PBMCs was 7.46 μM, which was substantially higher than that observed in Group A CHS cell lines and comparable to that in Group B. Notably, among the selected 19 compounds as Group B‐specific compound candidates, no compound was identified as Group B‐specific compounds based on the examinations of the IC_50_ value in each cell line.

**TABLE 3:** The 28 compounds selected as Group A‐ and Group B‐specific compound candidates in this study.

Compounds		Group A								Group B	
		CHS2	CHS4	CHS5	CHS6	CHS7	CHS8	DHS2	MHT2	DH82	DHS1
Group A‐specific	Duvelisib	+	+	+	+	+	+	+	+	−	−
	Erdafitinib	+	+	+	+	+	+	+	+	−	−
	Erlotinib	−	+	+	+	+	−	+	−	−	−
	Ivermectin	−	+	+	+	+	−	−	+	−	−
	Omadacycline tosylate	+	+	−	+	+	+	−	−	−	−
	Pemigatinib	+	−	+	+	+	+	−	−	−	−
	Pralsetinib	−	+	+	+	+	−	+	−	−	−
	Ripretinib	−	−	+	+	+	−	+	+	−	−
	Tivozanib	+	+	+	+	+	−	+	−	−	−
Group B‐specific	5‐fluoro‐5′‐deoxyuridine	−	−	−	+	+	+	−	−	+	+
	Amethopterin (R,S)	+	+	+	+	+	−	−	−	+	+
	Ancitabine hydrochloride	+	−	−	−	+	−	−	−	+	+
	Artemisinin	+	−	+	−	−	−	−	−	+	+
	Artesunate	+	+	+	−	−	−	−	+	+	+
	Cerivastatin	+	+	+	−	+	−	−	+	+	+
	Ciclopirox ethanolamine	+	−	+	+	+	−	−	−	+	+
	Cytosine‐1‐beta‐D‐arabinofuranoside hydrochloride	+	+	−	−	+	−	−	−	+	+
	Donafenib	−	−	+	+	+	−	−	+	+	+
	Everolimus	+	+	+	+	+	−	−	−	+	+
	Fingolimod	−	−	+	+	+	−	+	+	+	+
	Fluzoparib	+	+	−	+	+	−	−	+	+	+
	Fumagillin	+	+	+	+	+	−	−	−	+	+
	Nitisinone	−	−	−	−	−	−	−	−	+	+
	Posaconazole	−	+	+	−	−	−	−	−	+	+
	Protriptyline hydrochloride	−	−	+	−	−	−	−	−	+	+
	Quizartinib	+	+	+	−	+	−	+	−	+	+
	Triclocarban	−	+	+	−	−	−	−	−	+	+
	Trimetrexate	+	+	−	+	+	−	+	−	+	+

*Note:* +; The compound showed ≥ 30% of proliferation inhibition rate at 5 μM in the cell line, −; The compound showed < 30% of proliferation inhibition rate at 5 μM in the cell line.

### 3.2 Suppression of Akt Pathway by Duvelisib

**FIGURE 3:**
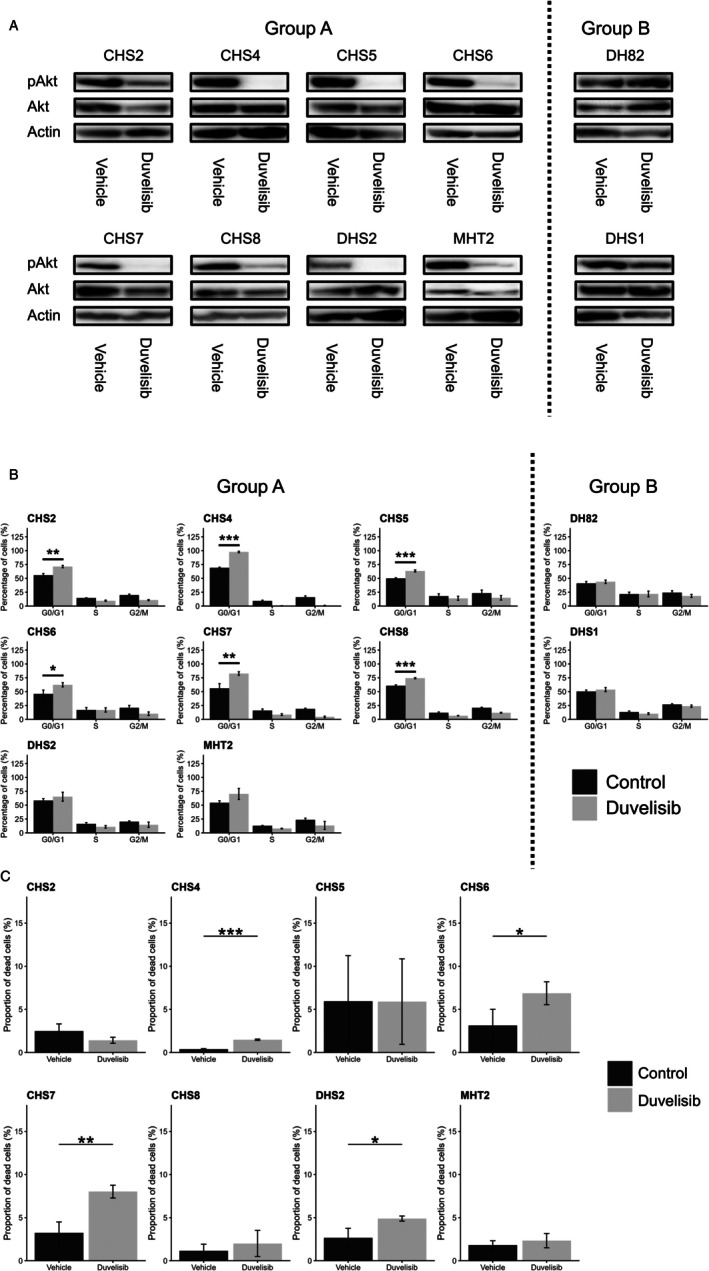
(A) Changes in the amounts of phosphorylated Akt protein in each canine histiocytic sarcoma cell line after treatment with 500 nM of duvelisib. (B) Changes in the distribution of cell cycle phases after treatment with 500 nM of duvelisib for 24 h. Black bars indicate the vehicle control group, and grey bars indicate the duvelisib‐treated group. *p < 0.05, **p < 0.01, ***p < 0.001. (C) Changes in the proportions of dead cells in each cell line after treatment with 500 nM of duvelisib for 48 h. All data are presented as the mean ± standard deviation from three independent experiments. Black bars indicate the vehicle control group, and grey bars indicate the duvelisib‐treated group. *p < 0.05, **p < 0.01, ***p < 0.001.

Duvelisib is a compound known to exert anticancer effects by inhibiting the activation of PI3Kγ/δ, leading to the suppression of the Akt signalling pathway in human cells [30, 31]. Thus, we evaluated its effect on Akt pathway activation in CHS cell lines. Proteins were extracted from CHS cell lines with or without treatment with duvelisib, and changes in the amounts of phosphorylated Akt protein were assessed by western blotting (Figure 3A). A decrease in phosphorylated Akt was observed in all eight Group A cell lines, whereas no change was detected in Group B cell lines, suggesting duvelisib specifically suppresses Akt activation in Group A CHS cell lines.

### 3.3 Evaluation of Cell Cycle Arrest and Cell Death Induced by Duvelisib

Akt signalling regulates cell cycle progression through increase of cyclin D [32]. Concurrently, Akt pathway activation inhibits apoptosis by suppressing pro‐apoptotic proteins [33]. Given that duvelisib inhibited the Akt pathway, we hypothesised that duvelisib treatment would lead to cell cycle arrest and the induction of cell death. Figure 3B shows the distribution of cell cycle phases following treatment with duvelisib. The proportions of cells in the G0/G1 phase significantly increased in six of the eight Group A cell lines, CHS2, CHS4, CHS5, CHS6, CHS7, and CHS8, and it tended to increase in MHT2, although it did not change in DHS2. In contrast, no significant change in G0/G1 phase distribution was observed in either of the Group B cell lines, DH82 and DHS1. As for the assessment of cell death, the proportion of dead cells was evaluated after treatment with duvelisib in Group A cell lines. An increase in cell death was observed in four of the eight cell lines: CHS4, CHS6, CHS7, and DHS2 (Figure 3C). These results suggested that duvelisib induces G0/G1 phase cell cycle arrest and cell death in a subset of Group A CHS cell lines.

### 3.4 Comparison of *PI3K* mRNA Expression Levels Between CHS Cell Lines

**FIGURE 4:**
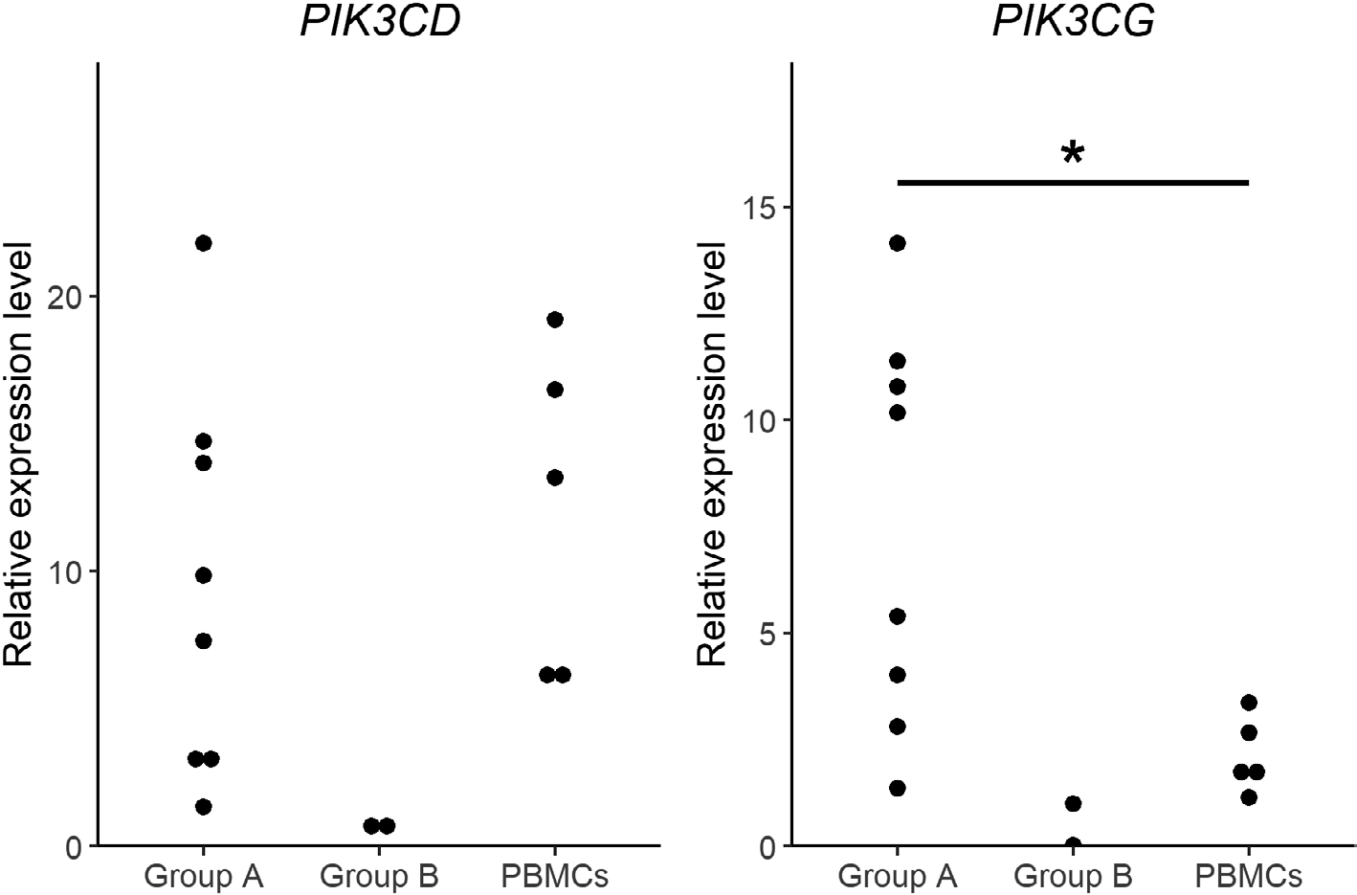
Relative mRNA expression levels of *PIK3CG* (encoding PI3Kγ) and *PIK3CD* (encoding PI3Kδ) in Group A, Group B, and peripheral blood mononuclear cells (PBMCs) isolated from healthy dogs. All data are presented as means of duplicates. *p < 0.05.

As a potential mechanism underlying the differential sensitivities to duvelisib among CHS cell lines, we hypothesised that the expression levels of PI3Kγ and PI3Kδ differ between the duvelisib‐sensitive Group A and ‐resistant Group B cell lines. We therefore compared the mRNA expression levels of *PIK3CG* (encoding PI3Kγ) and *PIK3CD* (encoding PI3Kδ) between Groups A and B, as well as with PBMCs. The gene expression levels of both genes were higher in Group A than in Group B, although statistical analysis was not feasible due to the small number of cell lines in Group B (Figure 4). When compared to PBMCs, *PIK3CG* expression level was significantly elevated in Group A, whereas *PIK3CD* expression level showed no significant difference between Group A and PBMCs.

### 3.5 Treatment of PI3Kγ Specific Inhibitor

**FIGURE 5:**
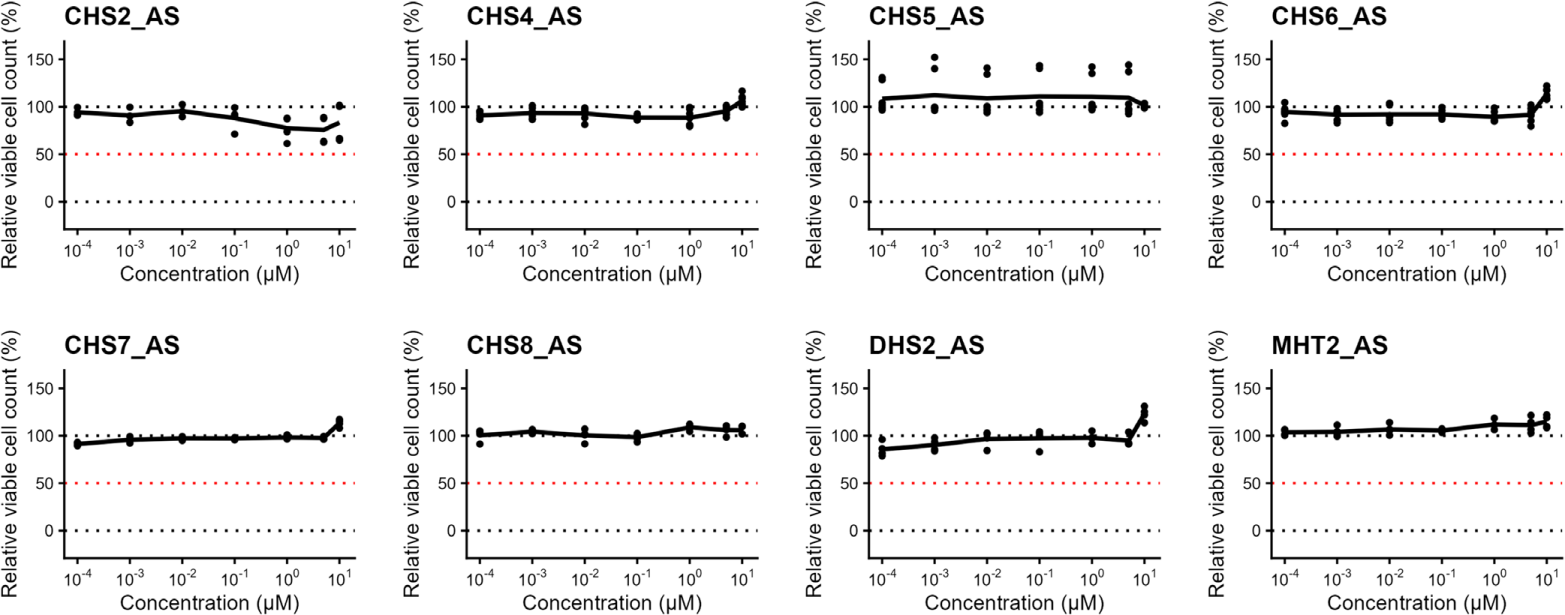
Dose–response curves illustrating antiproliferative effects of a PI3Kγ‐specific inhibitor (AS‐041164) in eight Group A cell lines.

Based on these findings, duvelisib was suggested to primarily target PI3Kγ, indicating that PI3Kγ may represent a potential therapeutic target in Group A CHS cell lines. Accordingly, AS‐041164 was administered to Group A CHS cell lines. However, treatment with the PI3Kγ‐specific inhibitor at a concentration of up to 10 μM did not suppress cell proliferation in any of the Group A CHS cell lines (Figure 5).

## 4 Discussion

The previous studies have shown that CHS is a tumour with considerable heterogeneity in drug sensitivity. In the present study, we performed HTS to identify compounds that exhibit specific antitumor effects against CHS cell lines of each molecular subgroup, Group A and Group B, classified based on gene expression profiles [19]. First, among the candidate compounds identified through HTS, 73 candidate compounds showed antiproliferative effects, reducing viable cell counts to ≤ 70% at 5 μM across all CHS cell lines. Besides these compounds, we identified 9 Group A‐specific compound candidates and 19 Group B‐specific compound candidates. Considering IC_50_ values of these compounds in each cell line, duvelisib was identified as one that selectively inhibited the proliferation of Group A but not Group B, while no compounds were found to specifically inhibit the proliferation of Group B cell lines. In Group A CHS cell lines, duvelisib was also shown to inhibit Akt pathway activation, leading to G0/G1 phase arrest and cell death in part of the cell lines.

The 73 compounds, which reduced viable cell count to ≤ 70% at 5 μM in all CHS cell lines, included anticancer drugs used in human medicine, such as 5‐fluorouracil and sunitinib [34, 35]. 5‐fluorouracil is an antimetabolite that inhibits DNA synthesis by incorporation into DNA during replication [36]. Sunitinib is a multi‐tyrosine kinase inhibitor, including vascular endothelial growth factor receptor (VEGFR) and platelet‐derived growth factor receptor (PDGFR) [37]. In the present study, we evaluated the therapeutic potential of these compounds for CHS by determining their IC_50_ values in the CHS cell lines. All 10 CHS cell lines exhibited IC_50_ values ≤ 100 μM for 5‐fluorouracil, and all values were below 700 μM, which corresponds to the reported *C*_max_ following administration at clinical dosage in healthy beagle dogs [29]. Therefore, this compound may represent a potential therapeutic candidate for CHS; however, further studies are needed to elucidate its mechanism of action and to evaluate its efficacy in vivo. In contrast, the reported maximum plasma concentration of sunitinib in humans is approximately 100 nM [28], and only two CHS cell lines exhibited IC_50_ values below this concentration in the present study, suggesting that sunitinib may have limited clinical applicability in CHS. It should be noted that the 73 compounds except for 5‐fluorouracil and sunitinib included those not used as drugs. For example, thonzonium bromide, a detergent component, was included in the 73 compounds. The antitumor effects of these compounds may arise from physical or chemical actions, such as disruption or dissolution of the cell membrane, suggesting their non‐specific cytotoxic effects. Since we could not examine whether the antiproliferative effects of these compounds were tumour cell‐specific, the effects of these compounds in normal cells need to be investigated in future studies.

The previous study has revealed the significant variabilities in drug sensitivities among CHS cell lines [18], and we recently showed that CHS cell lines could be divided into two major groups based on gene expression profiles [19]. These findings indicated that the effective chemotherapeutic agents should be selected based on the characteristic molecular abnormalities in CHS. Therefore, the purpose of this study was set to identify the compounds that specifically exert antitumor effects against each subgroup of CHS cell lines. In this study, we set the definitions of Group A‐ and Group B‐specific compounds subjectively, and we identified 9 Group A‐specific compound candidates and 19 Group B‐specific compound candidates. However, it is possible that more effective compounds against each subgroup were excluded during the selection process. For example, infigratinib, which was included in 287 candidate compounds, is a selective inhibitor of fibroblast growth factor receptor (FGFR), and clinical trials for various tumours in humans are currently ongoing [38]. Overexpression of FGFR1 has also been reported in CHS cases [13], and in the present study, infigratinib exhibited proliferative inhibition effects in all CHS cell lines except for CHS8. However, ponatinib, a multi‐kinase inhibitor targeting FGFR1 among other receptor tyrosine kinases, did not induce significant growth inhibition in most CHS cell lines in our previous study [13]. These findings suggest that the antitumor effects of infigratinib may not be solely attributable to FGFR1 inhibition and could involve FGFR1‐independent mechanisms.

Then, we assessed the dose‐dependent antiproliferative effects of the identified 28 Group A‐ and Group B‐specific compound candidates, and IC_50_ values for each compound were calculated in each CHS cell line. As a result, duvelisib was identified as the one with the greatest difference in IC_50_ between Groups A and B among the 28 compounds. Besides duvelisib, everolimus and trimetrexate exhibited low IC_50_ in all and eight CHS cell lines, respectively. Although we decided not to focus on these compounds based on the purpose of the present study, they are also candidates to be examined as novel therapeutic agents for CHS. Erdafitinib also demonstrated low IC_50_ values in Group A CHS cell lines, although not as low as duvelisib. Erdafitinib induces apoptosis by inhibiting FGFR activation as infigratinib and is currently being evaluated in clinical trials in humans [39]. Given the elevated FGFR1 expression reported in CHS [13], further exploration of the antitumor effects of erdafitinib may contribute to the identification of novel and more effective therapeutic strategies for CHS.

Duvelisib treatment induced inhibition of the Akt pathway signalling in Group A CHS cell lines, whereas it had no observable effect in Group B cell lines. The expected target of duvelisib is reported to be PI3Kγ or PI3Kδ, resulting in the inhibition of the Akt pathway, which has previously been shown to regulate CHS cell proliferation [18]. PI3Kδ is composed of the catalytic subunit p110δ and adaptor proteins, p85, p50, and p55, and it is activated by epidermal growth factor receptor, platelet‐derived growth factor receptor, and vascular endothelial growth factor rececptor [40]. PI3Kγ consists of the catalytic subunit p110γ and adaptor proteins, p101 and p84, and it is activated by G protein coupled receptors [40]. PI3K signalling is also known to regulate NF‐κB pathway activation [41], which was identified as a defining pathway in the molecular subclassification of CHS cell lines in our previous study [19]. Since differences in the activation status of NF‐κB pathway have also been observed among clinical CHS cases [42], its activation status may serve as a potential biomarker for selecting cases who may benefit from duvelisib treatment. Although the expression levels of both *PIK3CD* and *PIK3CG* were increased in Group A CHS cell lines compared to Group B cell lines, many PI3K inhibitors other than duvelisib, which were included in the compound library used in the present study, did not show antiproliferative effects in CHS cell lines. In a previous study, LY‐294002 and Wortmannin, inhibitors of PI3Kα, δ, and β inhibitors [43, 44], were administered to CHS cell lines; however, neither compound exhibited significant antiproliferative effects [17]. Therefore, PI3Kγ is considered the possible important target of duvelisib for CHS cell lines, and the differences in the amounts or activity of PI3Kγ might underlie the differences in the sensitivities against duvelisib between the groups. However, the specific inhibitor of PI3Kγ did not suppress cell proliferation. One possible explanation is that the inhibition of PI3Kγ was insufficient under our experimental conditions, as direct measurement of PI3Kγ activity was not feasible. Alternatively, duvelisib may exert its antiproliferative effects through dual inhibition of both PI3Kδ and PI3Kγ. It is also possible that duvelisib has additional molecular targets beyond PI3K that contribute to its antitumor activity. Moreover, the mechanism underlying the reduced expression of PI3Kγ in Group B CHS cell lines, as well as the precise mechanism by which duvelisib inhibits activation of PI3K isoforms, remains unclear and warrants further investigation.

The Akt pathway regulates the cell cycle through various downstream effectors such as glycogen synthase kinase 3 (GSK3), p21, p27, mechanistic target of rapamycin (mTOR), and tuberous sclerosis complex 2 (TSC2) [45]. Particularly, GSK3, p21, and p27 inhibit the activation of Cyclin D, thereby suppressing the phosphorylation of Rb protein and preventing cells in the G1 phase from progressing to the S phase [46, 47, 48]. In this study, it is presumed that suppression of Akt pathway activation through the inhibition of PI3K led to the activation of these proteins including p21, resulting in an increased proportion of G1 phase cells, in Group A CHS cell lines that showed cell cycle arrest by duvelisib treatment. However, the present study revealed that two of the eight Group A CHS cell lines did not show significant cell cycle arrest despite the inhibition of the Akt pathway. Actually, other proteins such as p16, p18, and p19 also inhibit Cyclin D activation [49, 50], and additional cell cycle related proteins such as Cyclin E are also involved in the G1‐S checkpoint [51]. The expressions and activities of these proteins are controlled not only by the Akt pathway but also by multiple intracellular signalling pathways, for example, NF‐κB and TGFβ pathways [52, 53]. Therefore, in Group A CHS cell lines where cell cycle arrest was not observed, it is possible that the activations of pathways other than the Akt pathway influenced the regulation of these cell cycle related proteins, thereby preventing cell cycle arrest. Future studies need to investigate the activation status of NF‐κB and TGFβ pathways in these cell lines.

Cell death includes multiple forms such as apoptosis, ferroptosis, and autophagy [54], and the Akt pathway is known to be involved in the regulation of these various modes of cell death [55]. Therefore, in this study, we evaluated overall cell death in a broad sense, rather than limiting the analysis to a specific death pathway. As a result, four of the eight Group A CHS cell lines exhibited the cell death induced by duvelisib, but the other four cell lines did not, which indicated that duvelisib inhibited the cell proliferations but did not induce cell death in these cell lines. It might also be explained by the associations of other intracellular signalling pathways than the Akt pathway. For example, the Akt pathway suppresses cell death by apoptosis through regulation of the Bcl‐2 family [55], but apoptosis can also be regulated via other pathways, such as the TNF receptor pathway [56]. Thus, it is possible that the death process was regulated by pathways independent of the Akt pathway in Group A cell lines where cell death was not induced. It remains to be clarified which pathways were involved in the regulation of cell death in these cell lines in future studies.

Common adverse events of duvelisib reported in human medicine include hematologic toxicities, such as anaemia and neutropenia, and gastrointestinal toxicities, such as diarrhoea and nausea [31, 57, 58, 59]. Although the adverse events in dogs remain to be examined, the IC_50_ of duvelisib in healthy canine PBMCs was 7.46 μM in this study, well above the plasma concentration of duvelisib in human medicine (approximately 2 μM) [31]. Furthermore, oral administration of duvelisib at 1.34 mg/kg in dogs resulted in *C*_max_ of 4.39 μM [60], These results indicate that plasma concentrations achievable in dogs are likely to exceed the IC_50_ values calculated for Group A CHS cell lines. Therefore, it is worth investigating the efficacies and toxicities of duvelisib in dogs with CHS.

Notably, most Group A CHS cell lines were derived from cases with solitary CHS [21]. Given that disseminated and solitary forms may exhibit distinct molecular pathophysiology, duvelisib may preferentially exert antitumor effects in solitary CHS.

Although several CHS cell lines used in this study harbour *TP53* gene mutations [61], *PTEN* deletions [62], and *PTPN11* mutations [63, 64, 65], all of which were previously identified in CHS cases, the association between these genetic alterations and duvelisib sensitivity has not been evaluated. Comprehensive genomic analyses, such as whole‐exome or whole‐genome sequencing, may help elucidate the correlations between duvelisib sensitivity and genomic aberrations and may facilitate the identification of predictive biomarkers.

In conclusion, we identified duvelisib as a therapeutic candidate showing antitumor effects specific to one of the CHS molecular subgroups defined by gene expression profiles. It is necessary to evaluate the efficacy and toxicity of duvelisib in CHS dogs, as well as to explore specific treatment strategies for the other molecular subgroup.

## Funding

This study was supported by the Japan Society for the Promotion of Science, KAKENHI (Grant Numbers 23K23778 and 23K05562) and JST SPRING (Grant Number JPMJSP2108), and this study was also supported by Basis for Supporting Innovative Drug Discovery and Life Science Research from AMED under Grant Number JP24ama121053 (support number 5139).

## Conflicts of Interest

The authors declare no conflicts of interest.

## Supporting information


**Figure S1:** Flowchart illustrating the selection process of candidate compounds using canine histiocytic sarcoma (CHS) cell lines. IC_50_, half‐maximal inhibitory concentration; HIT compounds, compounds that reduced viable cell count to ≤ 70% relative to vehicle‐treated controls; Group A‐specific, showing ≤ 70% viable cell counts in at least five Group A CHS cell lines and none of the Group B cell lines; Group B‐specific, showing ≤ 70% viable cell counts in five or fewer Group A CHS cell lines and in both Group B cell lines; ALL, showing ≤ 70% viable cell counts in all Group A and both Group B CHS cell lines.

**Figure S2A:** Dose–response curves illustrating antiproliferative effects of 5‐fluorouracil in each cell line.

**Figure S2B:** Dose–response curves illustrating antiproliferative effects of sunitinib in each cell line.

**Table S1:** Antibodies used in this study.

**Table S2:** Primer pairs used in this study.

**Table S3:** The 287 candidate compounds that reduced the viable cell count to ≤ 70% at 5 μM in at least two canine histiocytic sarcoma cell lines with reproducibility in this study.

## Data Availability

The data that support the findings of this study are available from the corresponding author upon reasonable request.
